# Major surgical conditions of childhood and their lifelong implications: comprehensive review

**DOI:** 10.1093/bjsopen/zrae028

**Published:** 2024-05-22

**Authors:** Paul S Cullis, Dina Fouad, Allan M Goldstein, Kenneth K Y Wong, Ampaipan Boonthai, Pablo Lobos, Mikko P Pakarinen, Paul D Losty

**Affiliations:** Department of Paediatric Surgery, Royal Hospital for Children Edinburgh, Edinburgh, UK; College of Medicine and Veterinary Medicine, University of Edinburgh, Edinburgh, UK; Department of Paediatric Surgery, Leicester Children’s Hospital, Leicester, UK; Department of Paediatric Surgery, Massachusetts General Hospital, Harvard Medical School, Boston, Massachusetts, USA; Department of Paediatric Surgery, Queen Mary’s Hospital, University of Hong Kong, Hong Kong, Hong Kong SAR; Department of Paediatric Surgery, Ramathibodi Hospital, Mahidol University, Bangkok, Thailand; Department of Paediatric Surgery, Hospital Italiano de Buenos Aires, Buenos Aires, Argentina; The New Children’s Hospital, Helsinki University Hospital and University of Helsinki, Helsinki, Finland; Department of Women’s and Children’s Health, Karolinska Institute, Stockholm, Sweden; Department of Surgery, University of Southern Denmark, Odense, Denmark; Department of Paediatric Surgery, Ramathibodi Hospital, Mahidol University, Bangkok, Thailand; Institute of Systems, Molecular and Integrative Biology, University of Liverpool, Liverpool, UK

## Abstract

**Background:**

In recent decades, the survival of children with congenital anomalies and paediatric cancer has improved dramatically such that there has been a steady shift towards understanding their lifelong health outcomes. Paediatric surgeons will actively manage such conditions in childhood and adolescence, however, adult surgeons must later care for these ‘grown-ups’ in adulthood. This article aims to highlight some of those rare disorders encountered by paediatric surgeons requiring long-term follow-up, their management in childhood and their survivorship impact, in order that the adult specialist may be better equipped with skills and knowledge to manage these patients into adulthood.

**Methods:**

A comprehensive literature review was performed to identify relevant publications. Research studies, review articles and guidelines were sought, focusing on the paediatric management and long-term outcomes of surgical conditions of childhood. The article has been written for adult surgeon readership.

**Results:**

This article describes the aforementioned conditions, their management in childhood and their lifelong implications, including: oesophageal atresia, tracheo-oesophageal fistula, malrotation, short bowel syndrome, duodenal atresia, gastroschisis, exomphalos, choledochal malformations, biliary atresia, Hirschsprung disease, anorectal malformations, congenital diaphragmatic hernia, congenital lung lesions and paediatric cancer.

**Conclusion:**

The increasing survivorship of children affected by surgical conditions will translate into a growing population of adults with lifelong conditions and specialist healthcare needs. The importance of transition from childhood to adulthood is becoming realized. It is hoped that this timely review will enthuse the readership to offer care for such vulnerable patients, and to collaborate with paediatric surgeons in providing successful and seamless transitional care.

## Teaser

In recent decades, survival of children with congenital anomalies and paediatric cancer has improved dramatically such that there has been a steady shift towards understanding their lifelong health outcomes. Paediatric surgeons will actively manage such conditions in childhood and adolescence, however, adult surgeons must later care for these "grown-ups" in adulthood. This article aims to highlight some of those rare disorders encountered by paediatric surgeons requiring long-term follow-up, their management in childhood, and their survivorship impact, in order that the adult specialist may be better equipped with skills and knowledge to manage these patients into adulthood.

## Introduction

Advances in fetal, neonatal, paediatric, anaesthetic and surgical care have led to increased survival rates in patients with congenital anomalies. The focus of patient care and research on decreasing the mortality rate of these index conditions has therefore dramatically shifted to reducing the associated morbidity rate. Many children born with congenital anomalies can now be expected to survive into adulthood. For some patients, the long-term effects of the condition may not significantly impact upon their lives. Nevertheless, for many complex conditions, adult survivors must navigate their lives affected by the physical, mental and psychosocial burden of their disease and its treatment. Paediatric surgeons operate on children with congenital anomalies and follow those patients with ongoing needs well into adolescence and young adulthood. As these children become young adults, their healthcare burden may be unmet without a successful, smooth transition to adult services, as some will require further or lifelong follow-up throughout their adult lifetime. Transitional care, which has been defined as ‘the purposeful, planned movement of adolescents and young adults with chronic physical and medical conditions from child-centred to adult-oriented healthcare systems’, is therefore of the utmost importance. This is a process which relies upon multidisciplinary team collaboration, in order to bridge the ‘care gap’ between paediatric and adult healthcare services^1,2^. Success requires enthusiasm, effort and collaboration between paediatric and adult specialists, to provide anticipatory, structured and multidisciplinary care. Once graduated from this process, the aims would be that the patient and their allocated adult clinicians will be equipped with the information and tools to provide holistic, ongoing follow-up into adulthood, which may be part of a formal adult healthcare programme. This article summarizes a range of major conditions which the paediatric surgeon manages through childhood, and which often require long-term, and in some cases, lifelong follow-up. The hope is to inspire and inform adult surgeons about diseases with which they have limited exposure yet can expect to encounter in increasing number in their surgical practice over subsequent decades.

A comprehensive literature review was performed to identify relevant publications in the English language published until October 2023, using MeSH headings and focused search terms relevant to each section. Several databases were employed, including PubMed, Embase, Cumulative Index to Nursing and Allied Health Literature, Cochrane, Web of Science databases and Google Scholar. Research studies, review articles and guidelines were actively sought, focusing on the paediatric management and long-term outcomes of surgical conditions of childhood. The study methodology was not planned to be a systematic review in accordance with PRISMA guidelines^3^.

## Upper gastrointestinal tract

### Oesophageal atresia and tracheo-oesophageal fistula

**Fig. 1:**
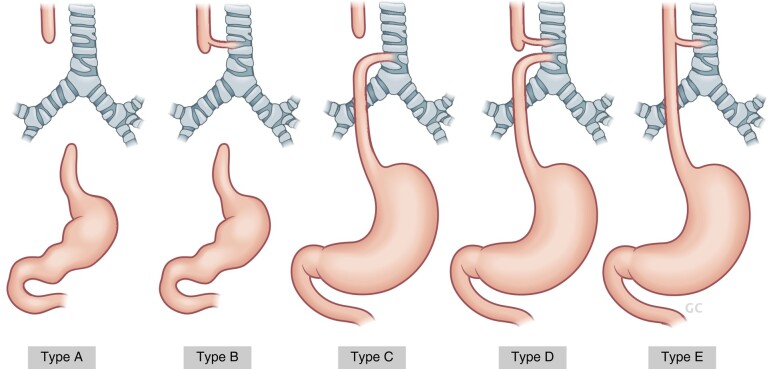
Gross classification system of the five subtypes of oesophageal atresia/tracheo-oesophageal fistula (OA/TOF)

Oesophageal atresia and tracheo-oesophageal fistula (OA/TOF) occurs in approximately 1 in 4000 births^4,5^. Most cases are sporadic, with less than 5% of cases occurring as part of a syndrome, such as Down (Trisomy 21) or CHARGE syndrome; however, approximately half of all patients have an additional congenital anomaly identified, which may be part of a recognized constellation of anomalies—the VACTERL association^5^. OA/TOF is considered a result of aberrant foregut development; however, the mechanisms responsible are poorly understood. The Gross classification is the most popular and divides OA/TOF into five subtypes, of which OA with a distal TOF accounts for 85% of cases (type C) and isolated OA without a TOF (type A) accounts for 8% (*Fig. 1*). In the former group, a primary anastomosis can usually be achieved at first surgery; in the latter configuration, there is typically a ‘long gap’ between the atretic segments such that delayed primary anastomosis, oesophageal lengthening techniques, gastric transposition or a graft interposition (for example colon or jejunum) are required. Since the first report of successful surgery in the 1940s, clinical outcomes of oesophageal atresia have dramatically improved, such that survival rates of 90–95% are possible in developed countries as a result of advances in neonatal, anaesthetic and surgical care. Nowadays the burden of complex co-morbidity is the major determinant of survival, such that infants with significant structural cardiac disease or extreme prematurity may still succumb^6,7^. Surgery can be performed by classical open thoracotomy, an axillary crease thoracotomy or thoracoscopic techniques, involving ligation of any TOF(s) followed by anastomosis of the upper and lower oesophageal segments.

Improvements in survival have unsurprisingly resulted in a growing number of adults living with OA/TOF. The past two decades has seen greater attention focusing on the lifelong implications of OA/TOF survivorship and its associated quality of life (QoL).

Dysphagia is a common symptom in patients with OA/TOF, affecting the majority of patients in the long term, yet is probably underreported as the innate differences in oesophageal motility which the patient has always encountered may not be fully appreciated as a symptom. Many use coping strategies, such as drinking liberal fluids with meals, dietary modifications and allowing additional time for meals^8,9^. New-onset or worsening dysphagia should be investigated promptly, so that a cause can be readily identified, such as oesophageal stricture; however, oesophageal dysmotility can itself result in swallowing difficulties. Food bolus impaction is a relatively common presentation in children with OA/TOF, yet seems to be less frequent in adults, perhaps due to the development of coping mechanisms to manage what many patients and families colloquially term ‘stickies’. Similarly, many patients experience regurgitation which persists into adulthood, and may represent oesophageal dysmotility or gastro-oesophageal reflux disease (GORD)^6^.

GORD is often present in children and adults with OA/TOF. When measured by pH-impedance studies or upper gastrointestinal (GI) endoscopy with biopsies, it affects approximately half of all patients yet many are asymptomatic^9–11^. Endoscopic evaluation is important as there is poor correlation between symptoms and presence of or severity of GORD. Antireflux surgery, notably Nissen fundoplication, has traditionally been undertaken in patients with OA/TOF; however, such operation(s) have become much less common with the availability of proton pump inhibitors (PPIs) and concerns of a wrap worsening dysphagia in the setting of an already dysmotile oesophagus. Unfortunately, there is no good evidence to suggest which, if any, antireflux surgery is best. Limited evidence suggests that complete wraps (for example Nissen) are more effective than partial wraps; however, these may cause worsening dysphagia, retching and gas-bloat symptoms. Conversely, partial wraps (for example Toupet or Thal) may carry less risk of these unwanted sequelae but seem to be less effective in treating GORD. Even with complete wraps, the failure rate may be high, as reported in limited series of adult patients undergoing antireflux surgery^12,13^. Over recent years, there have been growing concerns with regard to Barrett’s oesophagus and oesophageal adenocarcinoma in adult survivors of OA/TOF. Endoscopic studies have identified a four-fold increase in the prevalence of Barrett’s oesophagus amongst adults with OA/TOF compared with the normal healthy population. Gastric metaplasia is also notably more common. To date, there have been only a few reports of oesophageal cancer developing in patients with OA/TOF; however, the number of survivors into late adulthood is ever-increasing and this association will become clearer over the next few decades^14,15^. International paediatric gastroenterology guidelines recommend surveillance endoscopy with biopsies taken in four quadrants at the gastro-oesophageal junction (GOJ) and anastomosis every 5 to 10 years in adults with OA/TOF, having a low threshold for endoscopic assessment in the presence of new symptoms, and following adult consensus guidelines on metaplasia where present^16–20^.

In addition, eosinophilic oesophagitis appears more common amongst patients with OA/TOF than the general population, and treatment may be delayed due to misdiagnosis as oesophageal dysmotility or GORD. Representative biopsies at endoscopy are therefore essential to identify this entity as tailored treatment(s) improve symptoms and development of any stricture(s)^16^.

Oesophageal dysmotility is universal in OA/TOF. Abnormal manometric patterns are the norm amongst survivors, with reduced peristaltic amplitudes and uncoordinated or absent peristalsis, particularly of the distal oesophagus. Oesophageal emptying appears to be gravity dependent^6,8,11,16^.

Stricture at the anastomotic site is a common issue in OA/TOF, affecting nearly one in two infants within the first year of life^21^. Long gap OA and GORD increase the risk. Strictures are typically treated with balloon dilatation or bougienage and repeat procedures are common^22^. Refractory strictures may be managed by a variety of methods, including steroid injection or topical mitomycin C application, endoscopic incision or stent placement. Surgical resection or oesophageal replacement may be required in the rare instances where such measures fail. Strictures later developing in adult patients are unusual, and can suggest untreated GORD^23,24^.

Recurrent TOF occurs in less than 5% of patients after surgery for OA/TOF, being more common in the setting of excessive anastomotic tension and after anastomotic leak. New-onset cough, aspiration or recurrent chest infections are typical symptoms. This is, however, likely to be identified in childhood; few cases have been reported amongst the adult population^25^. Similarly, a missed TOF, particularly of the proximal oesophageal pouch, is also a possibility in patients with the aforementioned symptoms. Again, this is considered rare in paediatric patients, such that adult clinicians are unlikely to encounter it. Wider use of preoperative diagnostic tools (such as tracheo-bronchoscopy), greater awareness of a missed TOF by clinicians and therefore a lower threshold for targeted investigations should reduce their incidence and impact, however^26^.

The respiratory morbidity rate amongst OA/TOF survivors is increasingly recognized; however, the mechanisms by which it arises are again poorly understood. Young children with OA/TOF often experience tracheomalacia or airway collapse, managed by watchful waiting, positive ventilation strategies, and aortopexy or posterior tracheopexy surgery in some cases; however, this does not tend to be a major issue into adulthood. Adults may experience wheezing, regular cough, lower respiratory tract infections and episodes of bronchitis. It is important to exclude anatomical causes for new-onset symptoms (such as oesophageal stricture, recurrent or missed TOF, or vocal cord paralysis) although many will not have a cause identified. Studies, however, have generally identified mild reductions in pulmonary function testing, usually of a restrictive pattern, and exercise tolerance in most survivors is mostly considered unimpaired^6,27,28^.

Musculoskeletal problems after surgery for OA/TOF are common, such as scoliosis, chest wall deformity, winging of the scapula and shoulder dysfunction, rib fusion and breast disfigurement. Thoracotomy alone is responsible for many of these, and may be one major advantage of adopting minimally invasive approaches^15,29^.

QoL in adults with OA/TOF is often considered very good. That said, specific domains are affected more than others, particularly relating to the GI and respiratory system, as well as some patients experiencing reductions in psychosocial wellbeing. Long gap OA, revisional surgery, repeated hospital admissions and co-morbidities have a significant negative impact on QoL^6,7^.

Long gap OA may require oesophageal replacement with gastric transposition, jejunal interposition or colonic interposition. Such patients may experience a greater long-term morbidity rate than those with classical variant OA/TOF. There are inherent problems seen with each substitution graft, a subject widely reported in the literature^30^.

As a result of these lifelong sequelae, transition to adult services and close follow-up is essential for many adults born with OA/TOF ideally coordinated by clinicians with enthusiasm for this special patient group in a multidisciplinary clinic involving surgeons, gastroenterologists and/or respiratory physicians^10,12,16^. The International Network on Oesophageal Atresia has produced consensus guidelines for the transition of patients with OA/TOF which are a helpful resource for adult clinicians, as well as the aforementioned European Society for Paediatric Gastroenterology, Hepatology and Nutrition (ESPGHAN) and North American Society for Pediatric Gastroenterology, Hepatology and Nutrition (NASPGHAN) guidelines, and European Reference Network for Rare Inherited Congenital Anomalies (ERNICA) consensus document on OA/TOF^16,31,32^.

### Malrotation and midgut volvulus

**Fig. 2:**
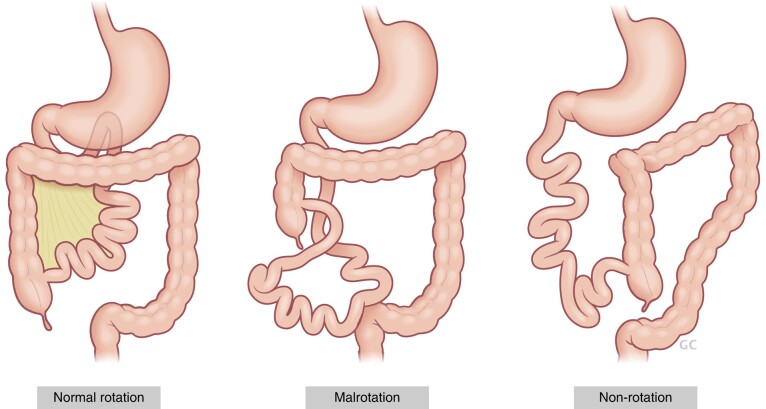
Variant anatomy of the midgut in three distinct morphologies: normal rotation, malrotation and non-rotation

Normal intestinal orientation is understood to occur in various stages. Physiological herniation of the embryonic intestine into the umbilical cord in the fourth week of gestation is associated with a 90-degree rotation. As the mid-gut returns to the abdominal cavity by the 10th week of gestation, an additional 180-degree rotation occurs. This is followed by a fixation process to the abdominal wall. This is the classic and simplified understanding of how the standard anatomical configuration of the bowel is achieved^33^. Intestinal rotational anomalies encompass a spectrum of congenital disorders whereby the normal embryological process of intestinal rotation and fixation are disrupted. At one end of the spectrum is non-rotation, where the small bowel lies to the right and the colon to the left of the abdomen cavity. This is, in fact, the orientation that is achieved by undertaking Ladd’s procedure, described later, and is often present when abdominal wall development is disrupted *in utero*, as in abdominal wall defects or congenital diaphragmatic hernia (CDH). The risk of volvulus here is typically considered to be low, but depends on the breadth of the mesentery and degree of fixation (which can only be convincingly demonstrated at laparoscopy or laparotomy). At the other end of the spectrum is the normal configuration of the bowel, where the duodenojejunal flexure lies to the left of the midline vertebral column in the upper abdomen, and the caecum fixed in the right lower quadrant. Between these two lie many possible variations, of which the most important clinically is malrotation. Malrotation refers to an orientation of the mid-gut which is liable to volvulus, in which the mesentery of the small bowel, centred around the superior mesenteric vasculature axis, is narrowed and therefore precariously at risk of volvulus. Malrotation is also commonly associated with Ladd’s bands, peritoneal attachments which tether the caecum to the posterior abdominal wall in the right upper quadrant, entrapping the proximal duodenum^34^. *Figure 2* illustrates these variant morphologies.

The true prevalence of these disorders is not known. It is conservatively estimated that symptomatic intestinal rotational anomalies are present in 1 in 5000 live births^33^; however, asymptomatic intestinal rotational anomalies occur much more frequently than this^35,36^. For example, 4% of the population have an atypical location of the appendix which is attributable to a mild rotational anomaly^37^.

Malrotation often presents in infants, but it can lead to symptoms at any age. The classical emergency presentation is of a neonate with bilious vomiting due to mid-gut volvulus; however, older children and adults can present with less typical symptoms, such as non-bilious vomiting, feed intolerance, chronic abdominal pain, early satiety, diarrhoea, failure to thrive and weight loss^38,39^. In one of the largest series, malrotation was diagnosed as frequently in adulthood as it was in childhood^38^. In paediatric practice, diagnosis is established by an upper GI contrast study, sometimes with ultrasound as an adjunct. Cross-sectional imaging can also demonstrate intestinal rotation; however, only laparoscopy or open surgery allows the surgeon to adequately assess intestinal fixation, or lack thereof. Surgery for malrotation typically involves a Ladd’s procedure, described by William E. Ladd in 1932. This traditionally comprises a laparotomy (usually by a transverse approach), detorsion of the mid-gut volvulus (if present) allowing reperfusion of the ischaemic bowel, division of Ladd’s bands, straightening of the duodenum loop, widening of the small bowel mesentery, replacement of the bowel in a non-rotated configuration, and often appendicectomy (by standard or inversion technique). Management of the asymptomatic patient is controversial, particularly in the setting of heterotaxy disorders, as a conservative approach may be appropriate in some, depending on co-morbidities, especially severity of cardiac anomalies, age, parental choice and the estimated lifetime real risk of volvulus^40,41^.

Adhesive bowel obstruction can affect a significant minority of patients after Ladd’s procedure, similar to small bowel surgery in adults. Studies with the longest follow-up demonstrate a risk of 10–20%^42,43^. Of interest, the use of laparoscopy is gaining popularity, particularly for patients in whom volvulus is not suspected. This appears to be associated with a lower risk of adhesive bowel obstruction; however, the risk of recurrent volvulus appears somewhat higher^43,44^.

Recurrent volvulus after surgery for malrotation with volvulus affects a minority of patients (less than 5%); however, it appears that minimally invasive techniques carry greater risk (3.5% estimate *versus* 1.4%). Less ‘helpful’ adhesions and inadequate widening of the mesentery have been implicated. Nevertheless, the risk of recurrent volvulus seems to reduce with advancing age, such that few case reports exist of adults developing recurrence^43^.

Many surgeons routinely perform appendicectomy at the time of surgery for malrotation; however, adult general surgeons should be aware that this is not universally the case, and the appendix may lie in an unusual location after a Ladd’s procedure. Appendicitis would be expected therefore to present with symptoms and peritonism focused on a location other than the right iliac fossa. The same may be true for gastroschisis, exomphalos and CDH, described elsewhere in this article.

Ischaemic necrosis of the small bowel due to mid-gut volvulus may lead to short bowel syndrome (SBS), described comprehensively in the following sections of this article.

A small proportion of patients in the long term may continue to have symptoms after surgery for rotational anomalies^45,46^. There appears to be a linear relationship between age at presentation and likelihood of postoperative symptoms^46^. That said, the majority of patients (more than 90–95%) experience resolution of their symptoms by having a Ladd’s procedure and it can be challenging in those experiencing symptoms to attribute these to their intestinal rotational anomaly.

It is important to note that, in the absence of significant loss of bowel due to volvulus, it would be unusual for a patient diagnosed with malrotation to require transition to adult services, but adult surgeons should be made aware that malrotation can present at any age.

### Paediatric short bowel syndrome

In SBS critical reduction of the gut length leads to intestinal failure (IF), characterized by severely impaired intestinal absorption and malnutrition requiring prolonged (more than 60 days) parenteral nutrition (PN) for survival and adequate growth^47^. Other important, but much less frequent, causes of IF in children are primary motility disorders such as paediatric intestinal pseudo-obstruction and congenital mucosal enteropathies^47,48^. According to population-based estimations, incidence of SBS among children is around 25 per 100 000 live births in developed countries^49,50^. As the most common underlying aetiologies of paediatric SBS include necrotizing enterocolitis (NEC) encountered in premature newborns, and congenital developmental disorders such as gastroschisis, intestinal atresia and mid-gut volvulus due to malrotation, the disease onset mostly takes place during early infancy^47,48^. While most children with SBS-IF eventually wean off PN with modern multidisciplinary care, those with less than 30% of age-adjusted remaining small bowel length, loss of ileum and ileocecal valve, and colonic resection may remain PN dependent for many years or permanently^51,52^. In selected children weaning off PN may have been supported by autologous intestinal reconstructive (AIR) surgery such as serial transverse enteroplasty (STEP) and longitudinal or spiral intestinal lengthening and tailoring^51,53^. Recent application of the enterotrophic glucagon-like peptide 2 analogue teduglutide therapy among children is likely to further promote achievement of enteral autonomy in paediatric SBS-IF^54^. Although ongoing developments in expert multidisciplinary management of IF have led to decreased rates of intestinal transplantation^55^, it remains a valuable rescue strategy for chronic IF in patients with life-threatening complications, while home PN remains a first-line therapy for paediatric IF^54^. Currently, overall long-term transplant-free survival of paediatric SBS-IF patients is estimated at over 95% in experienced tertiary centres^51,56,57^.

While patients with paediatric SBS-IF remain exposed to various nutritional, metabolic and other health issues, central venous access (CVA) problems, liver disease and different pathologies of the remaining bowel represent the most important life-threatening or surgically relevant long-term complications in paediatric SBS-IF^51,52,56^. With improved prognosis of intestinal failure associated liver disease (IFALD), central line complications have become a central cause for PN failure necessitating intestinal transplantation in paediatric SBS-IF^58,59^. Incidence of central line-associated bloodstream infections (CLABSI) is approximately 1–5/1000 catheter days in children with chronic IF^58^. As advised in the recent European position paper, in this patient group every febrile episode >38°C should be initially managed as potential CLABSI with central and peripheral blood cultures and empiric antibiotics while avoiding unnecessary central line removals to preserve precious CVA sites^58^. Prophylactic lock therapies with taurolodine or ethanol reduce CLABSI rates and should be at least considered for recurrent infections and high-risk patients with chronic IF^51,58^. Up to half of all paediatric IF patients develop central line-related thrombosis, which may lead to an already limited number of CVA sites at the time of transition to adult services^58^. In order to improve the longevity of CVA sites, catheter-related thrombosis should be treated with low molecular weight heparin and patients with recurrent thrombosis maintained on prophylactic anticoagulation^58^.

With improved understanding of the underlying pathophysiology, multidisciplinary management and advent of multisource fish oil containing parenteral lipids, progression of cholestatic liver injury to cirrhotic end-stage liver disease has become largely preventable in children with SBS-IF^51,57,59,60^. However, very premature highly PN-dependent newborns remain at increased risk^51,60^. In this respect, efficient prevention and treatment of central line and bowel-derived bloodstream infections is crucial as recurrent septic episodes promote cholestatic liver injury^60^. While cholestasis and portal inflammation predominate initial liver histopathology of IFALD, fibrosis and steatosis are similarly frequent findings during and after weaning off PN encountered at least in half of the patients^61^. Thus, continuous monitoring of hepatobiliary function primarily with serum liver biochemistry and measurement of liver stiffness as a surrogate of hepatic fibrosis should not be limited to PN-dependent patients, although pathophysiology and progressiveness of the fibro-steatotic form of IFALD remain currently unclear^60,61^.

In addition to small intestinal bacterial overgrowth (SIBO) and excessive adaptation-related small bowel dilatation, intestinal inflammation and anastomotic ulceration are increasingly recognized as clinically significant intestinal complications in children with SBS^52,62–65^. Excessive intestinal dilatation often coexists with mucosal inflammation associated with clinical symptoms of SIBO including diarrhoea, abdominal bloating and feeding intolerance, and this aggravates malabsorption^52,53,66^. Symptomatic dilatation resistant to antibiotic therapy may require tapering of an abnormally enlarged duodenal or small intestinal segment, not only to treat symptoms of SIBO by improving gut motility, but also to prevent liver injury by reducing bowel-derived bloodstream infections^52,53,66^. Although most patients wean off PN and experience improvement of their GI symptoms after AIR surgery, bowel dilatation and symptoms of intestinal dysfunction recur in a significant proportion of patients^52,53,67^. Intestinal inflammation and anastomotic ulcerations may cause intestinal bleeding, promoting recalcitrant iron deficiency anaemia, which may require parenterally infused iron supplementation^68^.

Management of anastomotic ulceration is challenging, and the diagnostic workup may require capsule endoscopy and enteroscopy in addition to routine upper and lower endoscopic examinations^65^. Although culture-proven SIBO often responds to oral antibiotics, it is associated with the presence of chronic intestinal inflammation resembling inflammatory bowel disease^63,64^. Medical therapy for both intestinal inflammation and anastomotic ulceration may be escalated from antibiotics, anti-inflammatory medications, corticosteroids to biologics based on severity of inflammation and the treatment response^63,65,69^. Other treatment options for anastomotic ulceration include endoscopic procedures, such as argon plasma coagulation and surgery, while recurrences are common after all treatment modalities and may later occur several years after initially successful therapy^65,69^.

Paediatric SBS predisposes to a multitude of complex morbidities and health problems which may manifest only after transition to adult care services and also in patients who have successfully weaned off PN prior to transition. Long-term outcomes of paediatric IF patients into adulthood have not yet been sufficiently studied or addressed and may markedly differ from those of adult IF due to an earlier disease onset at a vulnerable developmental period with higher cumulative disease duration and a largely different aetiology. For optimal continuation of care and outcomes, paediatric SBS-IF patients, including those who have weaned off after a prolonged period of PN, should be transitioned to a specialist interdisciplinary adult intestinal rehabilitation unit with access to intestinal transplantation. Kinberg *et al*. have published an informative review of the transition process for paediatric and adult clinicians^70^. Furthermore, there are many examples of adult care programmes for SBS globally, and several published guidelines for adult management^71,72^.

### Duodenal atresia

Duodenal atresia is a cause of duodenal obstruction in the neonatal period, occurring in between 1 in 5000–10 000 live births^73^. Although several theories have been proposed, embryologically the most accepted theory is a failure of recanalization of the duodenum lumen during the second month of gestation^74^. This can lead to a web, stenosis or complete atresia. External duodenal obstruction can also occur from developmental anomalies of surrounding structures leading to pancreatic anomalies, malrotation or Ladd’s bands^74,75^. There are three types of duodenal atresia: type 1 is a web or membrane causing an intrinsic obstruction, type 2 are two atretic portions of duodenum connected by a fibrous cord and type 3 is a complete separation of proximal and distal duodenal segments. It is important that if a type I duodenal atresia (web/stenosis) is identified during the surgery that a ‘windsock’ abnormality is ruled out: this is when the web and site of obstruction can extend more distally than the junction between the proximally dilated and distally collapsed duodenum.

Duodenal atresia usually presents in newborns with features of upper gastrointestinal obstruction; however, it should be noted that although the majority will have bilious vomiting or aspirates, as 85% of duodenal atresias have a postampullary obstruction, 15% are preampullary and these patients may therefore not have bilious vomiting or aspirates at presentation. The characteristic plain X-ray radiograph is that of a ‘double bubble’ and some babies may have had the index diagnosis suspected antenatally. It is well known that duodenal atresia can be associated with other anomalies, with over a quarter of patients having Trisomy 21 and half having another anomaly either in isolation (for example cardiac) or as part of an association (for example VACTERL association). Some children (for example those who have a duodenal stenosis) may present later in childhood^73,76^.

**Fig. 3:**
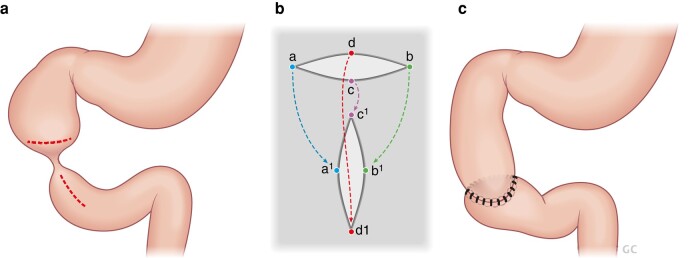
Kimura duodenoduodenostomy—a popular procedure for duodenal atresia

Surgery for duodenal atresia can be completed via an open or laparoscopic approach, with excellent outcomes achieved by both^77,78^. When surgery is performed via an open approach, this is usually via either a right upper quadrant transverse laparotomy incision or a transverse supra-umbilical incision. Various anastomoses and techniques have been described to bypass the duodenal obstruction; however, one of the most widely accepted is the diamond-shaped duodenoduodenostomy described by Kimura^79^ (*Fig. 3*). Some surgeons may perform a side-side duodenoduodenostomy, duodenoplasty, membrane resection or a duodenojejunostomy instead^76,80^.

Overall outcomes following repair of duodenal atresia are considered excellent, with postoperative survival rates of more than 95% expected. Death is usually related to associated anomalies rather than due to the duodenal obstruction^76^. A recent systematic review on the long-term outcomes of children who had correction of duodenal obstruction reported a 3% late mortality rate, and most of these deaths were due to cardiac anomalies or complications of cardiac surgery^81^.

Variable terminology for mechanical issues related to the bypass are described in the literature, including anastomotic dysfunction, stricture, duodenal diverticuli and megaduodenum. Overall, these collectively affect up to 5% of patients; however, without a strict definition, it is challenging to draw firm conclusions about the problem. Symptoms may include early satiety, bilious vomiting, halitosis and abdominal pain caused by eating. Upper GI contrast series and CT with oral contrast are helpful in diagnosis^81^. Balloon dilatation with or without use of endoscopic cautery has been reported; however, reoperation on with anastomotic revision may be required if symptoms are problematic and do not resolve with conservative management. The aforementioned and popular Kimura technique seems less prone to anastomotic issues. Blind loop syndrome is a rare problem which only occurs after less commonly employed bypass techniques (such as duodenojejunostomy) and can present with vomiting, early satiety, an abdominal mass or halitosis^81^. Moreover, in the few long-term series of duodenal atresia, peptic ulcer disease has developed in a very small number of patients; however, these historic series predate widespread use of PPI therapy^82^. A significant proportion of patients after duodenal atresia surgery have evidence of duodenogastric +/− biliary reflux on contrast and isotope studies, some of which develop reflux gastritis or duodenal irritation on endoscopic biopsies^83^.

Adhesive bowel obstruction is reported to occur in up to 5% of cases; however, follow-up in published series is limited, such that this is likely to be an underestimate of the true incidence. It is likely that laparoscopic surgery carries a reduced risk of adhesions; however, there is insufficient long-term data yet published^81^.

QoL in isolated duodenal atresia is generally comparable with peers. Patients with Down syndrome appear to have reduced social scores overall, but not in gastrointestinal domains^81,84^.

It is important to note that the literature on long-term outcomes of duodenal atresia is sparse; however, paediatric surgeons do not universally follow such patients into adolescence and the outcome in the long term is, in most, considered excellent. It would be unusual for a patient diagnosed with duodenal atresia to require transition to adult services.

### Gastroschisis

**Fig. 4:**
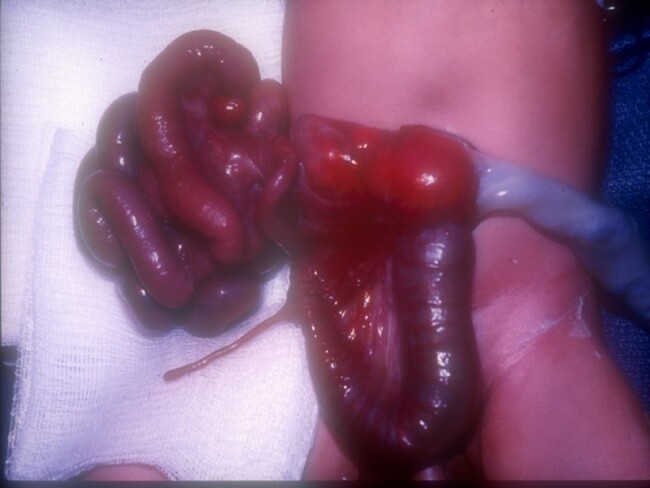
Newborn baby with gastroschisis

Gastroschisis occurs in approximately 1 in 4000 live births^85^ and is a congenital malformation where the abdominal viscera herniate through a right-sided abdominal wall defect with an intact umbilical cord and no covering membrane or sac over the organs (*Fig. 4*). This is usually an isolated malformation, so-called ‘simple gastroschisis’; however, 10–15% can be termed ‘complex gastroschisis’, where there is an associated atresia, ischaemia, necrosis, perforation or volvulus^86–89^. Several embryological theories as to how gastroschisis occurs have been proposed; however, its aetiology remains poorly understood^90^. Antenatal diagnosis is the norm. Following perinatal transition and stabilization, the goal is to return the herniated viscera into the abdominal cavity. This can be performed in newborns via early primary closure or a staged approach using a silo (soft plastic chimney) followed by delayed closure^91^.

Note herniated intestinal viscerae with umbilical wall defect to right of the cord structures.

The specific outcomes in gastroschisis are largely dependent upon the condition of the bowel at the time of closure. Overall long-term survival is 90–95%^87,92,93^. As expected, complex gastroschisis has higher morbidity and mortality rates^88,89^.

The major determinant of postnatal course is gut dysmotility and poor feed tolerance. This occurs because the bowel has been exposed to the biochemical effects of amniotic fluid *in utero*^94^. Newborns with simple gastroschisis take an average of 26 days to reach full enteral feeds and typically require some 28 days of total parenteral nutrition (TPN). For complex gastroschisis, these figures are 165 days and 90 days respectively^95^.

In the longer term, there is a moderately high risk of adhesive bowel obstruction in patients with gastroschisis, estimated to affect 25% of patients, with follow-up limited to childhood in almost all series. This risk appears to be highest in complex gastroschisis and within the first few years of life^96^. Patients are also more likely to develop chronic abdominal pain and constipation than the general population^96–98^.

Gastroschisis can cause SBS in a minority of patients with significant gut loss, as a result of a ‘closing’ or ‘vanishing’ gastroschisis, or from ischaemic complications after birth. It is estimated that just over 1% of patients develop SBS^95^, which has been comprehensively described previously.

Undescended testes are seen in approximately 20% of boys with gastroschisis at birth, and often the gonad(s) may be seen at primary defect closure. Half will descend spontaneously into the scrotum, otherwise, a single- or two-stage orchidopexy may be required in childhood. Success rates of surgery are lower than that of standard orchidopexy^99,100^. Therefore, adult survivors may have undergone orchidopexy, or experienced testicular atrophy during childhood.

Cosmesis is a major concern for some adult patients with gastroschisis, particularly where the umbilicus is absent, and plastic surgical interventions with umbilicoplasty may be appropriate in some cases^97^. Umbilical hernia is not infrequently seen in patients with gastroschisis, particularly after sutureless closure techniques; however, spontaneous resolution over time may occur. In those cases where closure does not occur, umbilical herniotomy is offered in later childhood, such that the adult surgeon is unlikely to be consulted for this issue^101,102^.

The long-term outlook for gastroschisis is usually considered excellent. QoL is generally considered comparable to the normal population in adult survivors with gastroschisis, despite some patients having concerns about cosmesis or gastrointestinal symptoms^97,103,104^. Similar to malrotation, in the absence of SBS, it would be unusual for a patient diagnosed with gastroschisis to require transition to adult services.

### Exomphalos

**Fig. 5:**
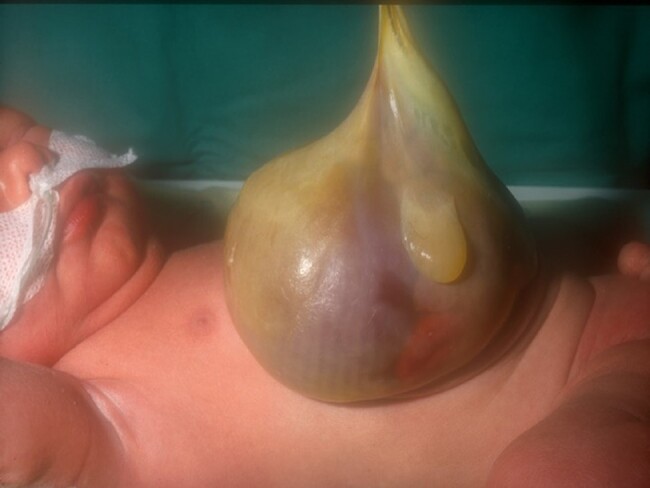
Infant with exomphalos major

Exomphalos occurs in 1 in 4000 live births and the majority are diagnosed antenatally^85^. It is characterized by herniation of the abdominal viscera with a covering membrane sac (*Fig. 5*). In contrast to gastroschisis, it has an association with other anomalies with greater than a third of babies having a chromosomal anomaly, such as Down syndrome or Beckwith–Wiedemann syndrome (BWS)^105,106^. Cardiac, renal and spinal anomalies can also occur. Exomphalos is often classified as minor or major, according to the defect size, which impacts upon primary management strategy^107^.

Note large abdominal wall defect containing liver covered with membranous sac.

The options for surgical management include primary, staged or delayed closure. Small defects are usually amenable to primary sutured closure. Larger defects can be closed primarily with use of a patch (biologic or synthetic); however, a staged approach is popular^107–109^. The sac itself can be used as a silo or the preformed silo deployed in gastroschisis can be used to allow gradual reduction of the viscera. Another option is to allow the sac to epithelialize before planning surgery in later infancy or beyond. Approximately a quarter of these patients will still require a patch at final repair and abdominal wall component separation surgical techniques are sometimes employed to permit definitive repair. There is a risk of recurrent ventral wall hernia^107,110^.

Patients with exomphalos do not tend to have as many gastrointestinal problems in the long term when compared with gastroschisis. Gastro-oesophageal reflux can be problematic. Gastrojejunal feeding may also be considered in this scenario. There is a high rate of children with exomphalos requiring placement of gastrostomy or gastrojejunostomy feeding devices in childhood, as well as those needing antireflux surgery for GORD, which can be technically challenging^105,111^. Similar to gastroschisis, there is a long-term risk of adhesive bowel obstruction (estimated at 5%) and mid-gut volvulus (estimated at 3%), which is a rare occurrence as intestinal rotation is normally arrested in a safe configuration^112^.

Similar to gastroschisis, boys with exomphalos have a higher incidence of undescended testes than the normal population. The likelihood of spontaneous scrotal descent is considered much less likely than that of gastroschisis, warranting orchidopexy. Adult surgeons may therefore encounter patients who have undergone surgery for cryptorchidism in early life^100^.

As mentioned, exomphalos is often associated with other anomalies and syndromes. Trisomy 21 and its health implications are well recognized amongst paediatric and adult clinicians alike, however, BWS (affecting approximately 10% of patients with exomphalos) is less frequently encountered and therefore not so well known amongst adult clinicians^113^. BWS is the commonest genetic overgrowth condition, with the typical features: macroglossia, macrosomia, postnatal overgrowth, exomphalos, organomegaly and urologic anomalies. It is a cancer predisposition syndrome, most commonly associated with Wilm’s tumour; however, hepatoblastoma, rhabdomyosarcoma, neuroblastoma, adrenocortical and Sertoli cell tumours can arise. The malignancy risk appears greatest in childhood; however, there are reports of adult-onset tumours. To date, there are no definitive international recommendations regarding cancer surveillance, even in childhood, but scheduling regular patient examination, blood tests (including α-fetoprotein level) and abdominal ultrasound is common practice until approximately 8 years of age^96,114,115^.

QoL in exomphalos appears to be considered generally good and comparable to peers. Nevertheless, approximately a third of patients with exomphalos will report ongoing intermittent abdominal pain that continues into adulthood and half are unsatisfied with the cosmetic appearance of their abdominal wall scar. This is particularly the case for those in whom the umbilicus has been sacrificed, that is in whom the sac has been completely excised^116^. It would be unusual for a patient diagnosed with exomphalos to require transition to adult services.

## Hepatobiliary

### Choledochal malformations

**Fig. 6:**
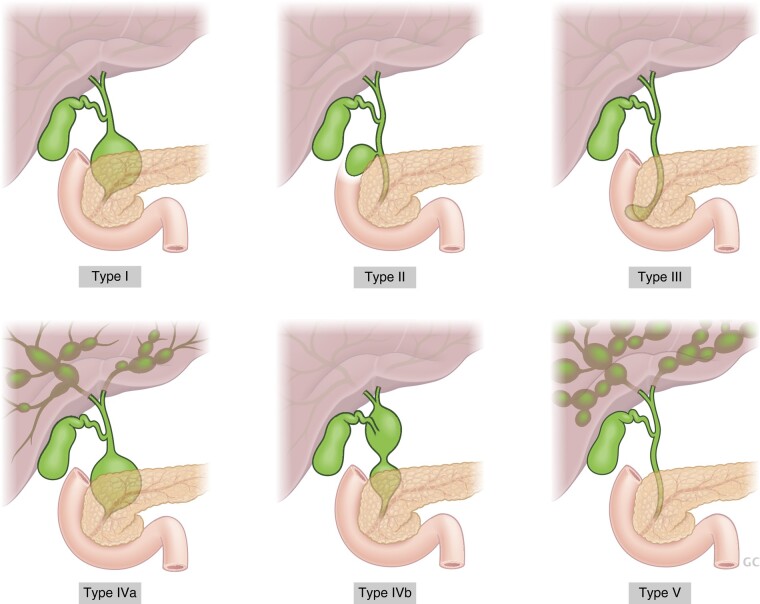
Todani classification divides choledochal malformations into five distinct groups: type i (80–90%)—dilatation only involving the extrahepatic bile duct, type II (3%)—a diverticulum of the extrahepatic bile duct, type III (5%)—a choledochocoele which involves dilatation of the intraduodenal part of the common bile duct, type IV (10%)—dilatation involving both the intra- and extrahepatic bile ducts, and type v (rare)—Caroli’s disease

Choledochal malformations (CMs), formerly known as choledochal cysts, are congenital biliary tract anomalies with abnormal dilatation of the hepatobiliary tree. The morphological classification of CMs was first proposed by the Japanese surgeon, Takuji Todani^117^, whose classification is widely adopted by the international surgical community (*Fig. 6*). CMs occur more commonly in females (3–4:1) and the estimated incidence is much higher in Asia (1:1000) than in Europe (1:37 400)^118,119^. The majority of CMs become symptomatic and are treated during childhood^120^. Typically, infants present with painless jaundice whereas older children may experience recurrent abdominal pain which can be secondary to cholangitis or acute pancreatitis, while around 15% are now detected on antenatal ultrasound.

The aetiology of CM remains debatable. The two leading theories involve either the presence of an anomalous pancreatobiliary junction (PBM) with associated reflux of pancreatic juice into the biliary system or congenital biliary obstruction with resultant pressure-driven proximal bile duct dilatation^118^. In PBM, the choledochal sphincter is located distal to the junction of the common bile duct and the pancreatic duct forming an abnormally long common channel. As a result, digestive pancreatic enzymes may reflux freely into the bile ducts, resulting in inflammation, tissue destruction, dilation and cancer (the Babbitt hypothesis)^121,122^.

CMs require timely surgical excision as, if left untreated, they can lead to various major complications, including obstructive cholestasis, bile duct perforation, recurrent cholangitis, pancreatitis and development of cholangiocarcinoma. In symptomatic children, surgery is performed as soon as the patient’s clinical condition allows. Many cases are, however, diagnosed in asymptomatic children. Surgery is typically planned in such cases on an elective basis^123^. A wide variety of imaging modalities are available for assessing CMs, while most lesions are primarily diagnosed by ultrasound examination. Additional preoperative imaging is aimed at outlining the biliary tree, identifying coexistent stone disease, and delineating the anatomy of the PBM for safe removal of the intrapancreatic portion of the common bile duct as distally as possible while avoiding pancreatic duct injury. In addition, intraoperative cholangiography and choledochoscopy for thorough clearance of retained stones and debris are routinely used. Although CT scan and 3D-reconstructed MRI have been used increasingly for more precise preoperative delineation of CM morphology, up to 40% of PBMs are still missed with these imaging modalities^124^. Further assessment using endoscopic retrograde cholangiography, percutaneous transhepatic cholangiography and temporary biliary stenting before surgery may be employed in complicated cases^120^.

Due to the risk of malignant degeneration, surgical management of CMs involves complete excision of the extrahepatic biliary tract combined with biliary-enteric reconstruction. Currently used options to restore biliary-enteric continuity include hepaticojejunostomy with Roux-en-Y reconstruction, and hepaticoduodenostomy, which are actively debated. Classic open hepaticojejunostomy has been widely advocated and is still used by the majority of European paediatric surgeons^124^. An increasing trend and preference towards minimally invasive and robotic surgery, especially in Asian centres, has driven hepaticoduodenostomy as an alternative method of biliary-enteric reconstruction. In this respect, it offers several advantages including technical ease of a single anastomosis and shorter operative time with postoperative endoscopic access to the anastomosis should a stricture or stones develop, while predisposing to bilious gastritis, reflux of pancreatic enzymes into intrahepatic bile ducts and a longer remnant of the common hepatic duct^125^. Comparable outcomes following both biliary-enteric reconstructions have been reported^126–128^, although cumulative long-term experience is much larger with hepaticojejunostomy. For a narrow common hepatic duct in younger children, hepatic ductoplasty with hepaticojejunostomy is preferred to widen the biliary-enteric anastomosis and prevents stricture when the ductal size is considered small^129^.

The main long-term complications of CMs include cholangitis, stone formation, stricture of the biliary-enteric anastomosis and malignancy^125^. Cholangitis, which usually responds to antibiotic therapy, is reportedly the most common long-term complication after CM surgery in children with an incidence of 13–20% recorded, the risk being particularly high in type IV CMs^125,130^. Especially when recurrent cholangitis or hepatolithiasis occurs after CM surgery, a biliary-enteric anastomotic stricture should be ruled out with magnetic resonance cholangiopancreatography (MRCP). A verified anastomotic stricture is primarily managed with endoscopic or transhepatic balloon dilation with a high success rate, while reoperation on is considered as a secondary option^131^. Tsuchida *et al.*^132^ described variant types of intrahepatic bile duct (IHD) anatomy associated with postoperative intrahepatic cholelithiasis. In their study, these were classified into three types, i.e. type I, no IHD dilation; type II, IHD dilation without downstream stenosis; and type III, dilatation of IHD with downstream stenosis. Intrahepatic cholelithiasis developed only in 1% of patients who had types I and II IHD dilatation. In contrast, for type III IHD dilatation the occurrence was as high as 40%. The reported time interval after surgery to the development of intrahepatic stone disease ranges anywhere between a few years to several decades.

The risk of biliary malignancy in adult patients with CM ranges from 6 to 30% with typical age of presentation at 45–51 years^125,133–135^. According to a recent meta-analysis, 1.8% of patients with CM type I or type IV developed malignant transformation after complete cyst excision^136^. The reasons for the malignant transformation following cyst excision are unclear and it may develop anywhere in the remaining parts of the biliary tree. Biliary dysplasia is found in some 15% of resected CM specimens, while postoperative MRCP may demonstrate an excessive distal remnant of the common bile duct in occasional patients mandating intensified follow-up or excisional surgery^120,134,137^. Incomplete excision of the distal cyst or PBM may also lead to protein plug formation and postoperative pancreatitis, which is a rare complication^125^. In addition to postoperative adhesive obstruction and volvulus, other possible factors of intestinal morbidity rate linked to CMs include stricture of the enteroanastomosis, retrograde intestinal intussusception as well as inflammatory bowel disease^137,138^.

Patients operated on for CM in childhood are at risk of developing cholangitis, stricture of the biliary-enteric anastomosis, hepatolithiasis and biliary malignancies. As the fate of these potential long-term complications remains currently unclear, based on the current evidence different authors and institutions are increasingly committed to structured follow-up programmes, which continue beyond childhood with transition to adult care^125,139^. Nevertheless, as with many of the conditions discussed in this review, transitional care models and adult follow-up programmes remain in their infancy. Few recommendations have been published to date^140,141^.

### Biliary atresia

**Fig. 7:**
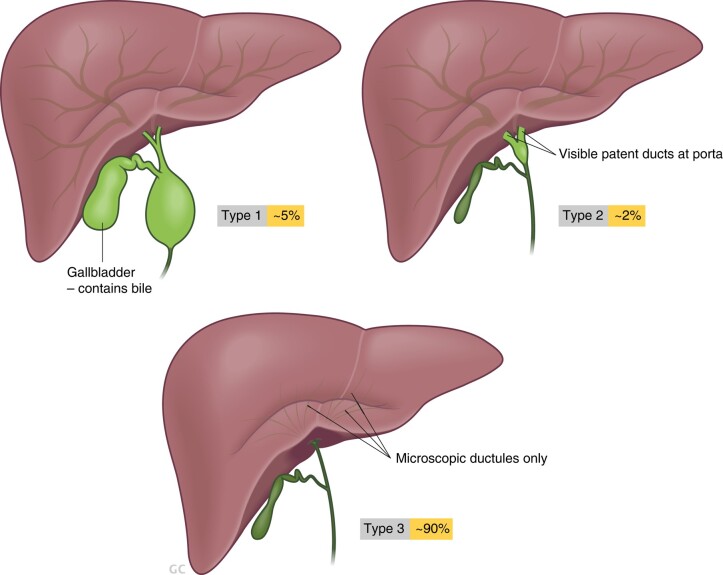
**Schematic illustration of the three major subtypes of biliary atresia (adapted from Hartley *et al.*^142^**)

Biliary atresia (BA) is a congenital disease characterized by progressive fibrosclerosing obliterative cholangiopathy. It is the leading cause of obstructive jaundice in neonates and the most common indication for liver transplantation in children worldwide^142^. The prevalence of BA varies by geographic region and race with an incidence of 1 in 5000 to 8000 live births in Asia and around 1 in 14 000 to 19 000 live births in Western countries^143^. The underlying reason for the discrepancy may be due to differences in population genetics and predisposition to environmental factors between the East and West. Several candidate genes, such as *ADD3* and *GPC1,* have been shown to associate with BA^144^. A viral trigger causing biliary obstruction *in utero* has long been considered a possible aetiological factor. Viruses implicated in BA include cytomegalovirus (CMV), reovirus and rotavirus. CMV is the most frequently analysed virus with a possible connection to poor surgical outcome^145^. Furthermore, as BA patients exhibit a dominant Th1 immune response, and other studies have demonstrated a decrease in frequency and function of T regulatory cells (Tregs), immune dysregulation seems an important factor in the pathogenesis of BA^146,147^. BA can be categorized into several clinical subtypes: isolated BA (IBA), syndromic BA (SBA), cystic BA (CBA) and cytomegalovirus-associated BA (CMVBA), reflecting aetiological heterogeneity of the disease^148^. It can also be categorized according to the level of the most proximal obstruction (*Fig.* 7)^142^.

**Fig. 8:**
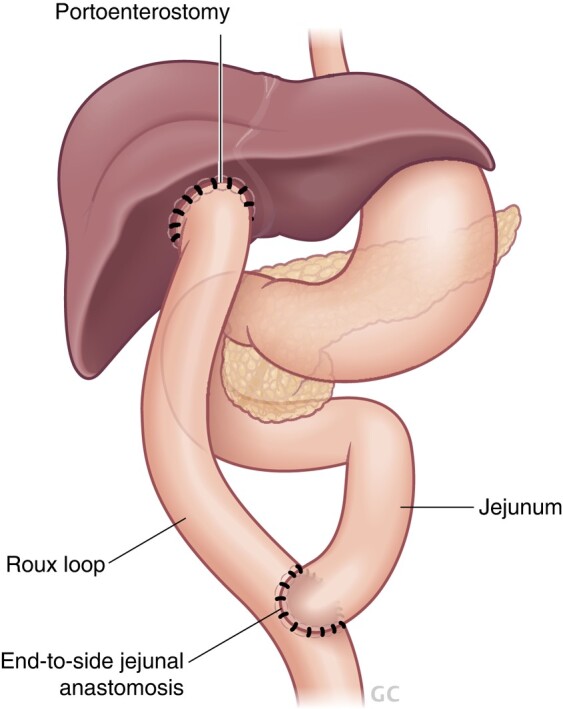
The Kasai portoenterostomy

For the diagnosis of BA, intraoperative cholangiography remains the ‘standard’. Once confirmed, Kasai portoenterostomy (KPE) is performed to restore adequate biliary drainage. KPE was first described by Morio Kasai in 1959 with the excision and transection of the obliterated extrahepatic bile ducts at the level of the portal plate, followed by drainage of remnant microscopic bile ductules using a Roux-loop (*Fig. 8*)^149^. Despite this revolutionary procedure, the resolution rate of cholestasis and long-term native liver survival remain suboptimal, and most patients still require liver transplantation before reaching adulthood^150–155^. In patients who present late with advanced cirrhosis, primary liver transplant without prior KPE is considered the best treatment option.

After excision of the extrahepatic biliary tree, the porta hepatis is transected to expose the remaining microscopic ductules and a jejunal Roux limb is anastomosed to the transected surface.

Clinical outcome after KPE remains difficult to predict due to disease heterogeneity and contribution of multiple clinical variables. The age at KPE has long been identified as one of the main factors predicting the likelihood of resolution of cholestasis. As the KPE performed before 60 days of life has been shown to improve native liver survival, early diagnosis and treatment is vital. Patients who fail to normalize their serum bilirubin by 3 months after KPE are likely to require liver transplantation by the age of 2 years^156,157^. Individual centres’ expertise and BA subtype also impact patient outcome^158^. Patients with evidence of CMV infection may have poorer KPE outcomes and some centres now administer antiviral therapy for this specific group of patients^159,160^. After surgery, the use of steroids is widely adopted for its anti-inflammatory effect and promotion of bile flow. A recent RCT and meta-analysis both confirmed that postoperative adjuvant steroid therapy improved bile drainage and native liver survival^161,162^. Recurrent cholangitis is another significant factor which negatively affects native liver survival. Thus, post-KPE patients who present with fever and cholangitis should be promptly treated with antibiotics^163,164^.

Management following KPE includes prevention and treatment of chronic liver disease complications and optimization of nutrition and growth. Failure to establish adequate bile flow after KPE leading to rapidly progressing liver failure, intractable cholangitis and growth failure are common indications of early liver transplantation post-KPE. Overall, about half of BA patients after KPE require a liver transplant by 2 years of age. The outcome of BA patients after liver transplantation is generally excellent, with graft and patient survival reaching 90 and 97% respectively^165^.

Patients who continue to survive with their native liver require lifelong follow-up. Eventually most of these patients develop complications of chronic cholangiopathy, including cirrhosis, portal hypertension with oesophageal varices, and recurrent cholangitis^150,166^. As well as these liver-related problems, native liver survivors are predisposed to impaired bone health, neurodevelopmental problems and reduced QoL^166^. BA patients with disease complications report a significantly lower vitality score than those without^167^. Similar findings were found in a recent systematic review summarizing nine QoL studies^168^. For female survivors who reach adulthood, pregnancy has been associated with serious complications^169^. Transition for BA patients from paediatric to adult services may be challenging. Adult health professionals may have limited experience with the condition-specific potential complications, both medical and psychosocial. For example, adult liver disease scoring systems are not optimal tools to predict the need for liver transplantation in adult BA patients^170^. Setting up multidisciplinary transition clinics attended by both paediatric and adult professionals promotes continuation of efficient patient care^141^. Nonetheless, as before, transitional care models and adult follow-up programmes remain in their infancy. Few recommendations have been published to date but this is an evolving area of research interest^140^.

## Colorectal

### Hirschsprung disease

Hirschsprung disease (HSCR) is a congenital condition in which neural crest-derived cells fail to complete their rostrocaudal migration along the length of the intestine, leaving the distal bowel devoid of ganglion cells^171^. The length of aganglionosis is variable, with 75% of cases limited to the rectosigmoid (referred to as short-segment HSCR), approximately 8% having total colonic aganglionosis and 1% exhibiting total intestinal aganglionosis^172^. HSCR affects 1 in 5000 births, with a male-to-female ratio of 4:1, and 8% having Down syndrome^173^. HSCR is a polygenic disorder with variable inheritance patterns and rearranged during transfection (RET) mutations being the most common genetic aetiology^173^. Most cases present in neonates, with failure to pass meconium within the first 48 h of life. This, often combined with abdominal distension, emesis and feeding difficulty, prompts a contrast enema that may show the classic finding of a narrowed rectum with proximal colonic dilatation. The ‘standard’ for diagnosis is a rectal biopsy, typically done at the bedside, which will show the absence of ganglion cells, loss of calretinin immunostaining and the presence of hypertrophic nerve fibres^171,173^.

Once the diagnosis is made, rectal irrigations are initiated to evacuate stool and thereby decompress the colon^173,174^. For short-segment HSCR, definitive pull-through surgery is typically performed within the first few months of life. In children with more extensive aganglionosis, irrigations are often ineffective and a diverting ostomy is required. In these cases, stoma reversal and pull-through surgery are timed individually^171,172^.

**Fig. 9:**
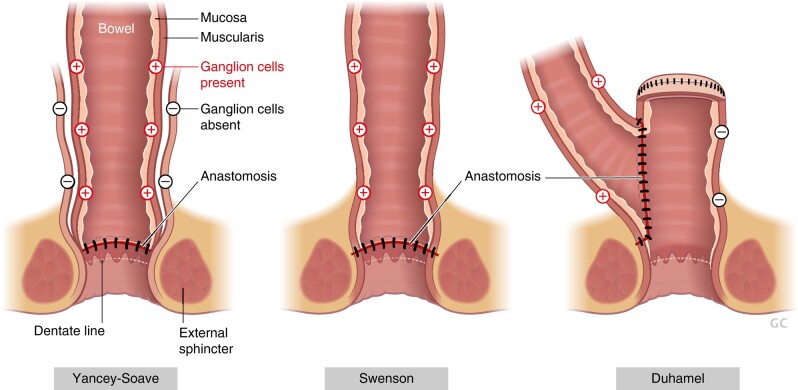
Schematic illustration of the three common operations for Hirschsprung disease—Yancey-Soave, Swenson and Duhamel procedures

The fundamental principle of operative treatment for HSCR is to bring normally ganglionated bowel to the anus. The most common operations performed include the Swenson, Yancey-Soave and Duhamel procedures (*Fig. 9*)^175^, each of which is an effective approach, the choice largely depending on the surgeon’s preference^171,173^. In the Yancey-Soave pull-through, the muscular wall of the aganglionic rectum is left in place, and the more proximal ganglionated bowel is pulled through this muscular cuff and anastomosed to the anus. This is done to avoid dissection around the rectum and injury to the pelvic innervation. This operation is commonly performed transanally with laparoscopic assistance both to obtain frozen section biopsies of the bowel and to help with mobilization of the colon and division of the mesentery. In contrast to an endorectal pull-through, the Swenson procedure involves a full-thickness dissection of the rectum down to the anal canal. Another common operation for HSCR is the Duhamel procedure, in which a retrorectal dissection is performed and the distal aganglionic rectum is preserved. The proximal ganglionated bowel is brought down behind the rectum and a side-to-side anastomosis performed, thereby creating a neorectum that can function as a reservoir for stool and which is composed of normally innervated bowel posteriorly and aganglionic rectum anteriorly. In each of these operations, it is important to obtain intraoperative biopsies for frozen section analysis of the proximal margins to confirm that all the aganglionic bowel is removed. In addition, the transition zone, which refers to the hypoganglionic segment between the aganglionic and normal bowel, also needs to be excised and removed, which is why an additional 5–10 cm of bowel is resected proximal to the biopsy confirming the presence of ganglion cells^171^. Patients with total or near total intestinal aganglionosis are managed with end-jejunostomy and permanent parenteral nutrition, which may necessitate intestinal transplantation later in life^176^.

N.B. ‘+’ symbol denotes the presence of ganglion cells; ‘–’ symbol indicates absence of ganglion cells. Adapted from Dasgupta and Langer^175^.

Despite removal of the aganglionic segment, children with HSCR are at risk of long-term complications following surgery^171,173,177^. These include impaired faecal control, obstructive bowel symptoms and Hirschsprung-associated enterocolitis (HAEC). The reasons for these are not entirely known, but a multitude of factors have been proposed, including abnormal motility of the remaining bowel, non-relaxing anal sphincters, dysbiosis, iatrogenic injury to pelvic innervation or anal sphincters, and functional constipation^171,173,177,178^.

HAEC is the most common cause of death in HSCR. It is an inflammatory condition that is associated with obstructive symptoms and presents with foul-smelling diarrhoea, abdominal distention, vomiting and fever, sometimes progressing to sepsis^171,173,178^. Its aetiology is unknown, but appears to be multifactorial, including abnormalities within the enteric immune system, epithelial barrier integrity and intestinal microbiome^171,173^. HAEC can occur before or after definitive surgery for HSCR and its reported incidence is highly variable, ranging from 10 to 40%, due to lack of a clear definition or any diagnostic clinical markers. Treatment for mild cases includes rectal irrigations and oral metronidazole, while more serious cases require inpatient management with intravenous fluids, broad-spectrum antibiotics and rectal irrigations. Diverting ileostomy can be lifesaving for severe, refractory HAEC. Fortunately, HAEC most often stops occurring by the time a child reaches school age^179^.

Obstructive symptoms refer to severe difficulty evacuating, usually with concomitant abdominal distension^150,178^. This is relatively common, occurring in up to 33% of patients, and can persist into adolescence and adulthood^177^. Multiple causative factors have been identified, including: mechanical obstruction of the bowel due to anastomotic stricture, twisting of the pull-through, a tight muscular cuff following a Yancey-Soave endorectal pull-through or rectal spur after a Duhamel pull-through; internal anal sphincter achalasia, since the normal recto-anal inhibitory reflex is absent in HSCR, even after pull-through surgery; a transition zone pull-through, in which the abnormally innervated transition zone is not removed; colonic dysmotility due to abnormal enteric neurons even in the seemingly normal ganglionated bowel; and functional constipation, in which children develop withholding behaviour due to a history of anal surgery and painful defaecation^171,173,178,180^. The evaluation of a patient with obstructive symptoms can include anorectal examination, contrast enema, repeat rectal biopsy, anorectal manometry and colonic manometry, with the evaluation tailored to the case. It is very important to establish the cause so that a targeted treatment strategy can be initiated^180^.

Faecal soiling (escape of small amounts of liquid or soft stool) is a common long-term complication, affecting up to 50% of adult patients after surgery for HSCR^172,181^. Soiling is often associated with an increased defaecation frequency due to active peristalsis in the pulled through bowel and the absence of a normal rectum to serve as a reservoir for stool, often also with the inability to feel the urge to defaecate^177,178^. True incontinence, with inability to hold back defaecation, is due to abnormal anorectal sensory function or inadequate sphincter control, and is treated with bowel management, including retrograde or antegrade enemas, or a diverting ostomy^182^. In contrast, patients with faecal impaction with resulting overflow incontinence should be considered in the category of obstructive symptoms and therefore the evaluation should be as described above^178,179,182^. While faecal control usually improves with age, especially around adolescence^179,183^, variable degrees of faecal incontinence or constipation, depending on the type of pull-through, persist into adolescence and adulthood in 30–50% of patients^177,179,183,184^. This has a significant impact on QoL as diminished faecal control correlates highly with social, emotional and sexual wellbeing^183,185,186^. Interestingly, while bowel function often improves with time, some aspects of QoL may not, possibly reflecting residual psychological burden from childhood^185^.

Although rectal dissection predisposes to iatrogenic pelvic injuries, lower urinary tract and erectile function appear well preserved in the majority of adult HSCR patients^186–188^. However, adult female patients not only reported decreased sexual QoL, but also difficulties conceiving spontaneously, possibly related to postoperative pelvic adhesions^188^. Another increasingly recognized and poorly understood long-term complication of HSCR is inflammatory bowel disease. In a Swedish national study, the risk of inflammatory bowel disease, mostly classified as Crohn’s disease, was about five-fold higher than in the general population^189^. Some rare RET mutations causing HSCR may also give rise to familial medullary thyroid carcinoma, which usually manifests in adulthood^190^.

Transitional care pathways and adult care programmes for congenital anorectal disorders are not widely adopted^174,191,192^. The authors are not aware of specific adult guidelines for patients with Hirschsprung disease; however, the ERNICA guidelines for the management of rectosigmoid Hirschsprung disease do include recommendations for adult follow-up^171^.

### Anorectal malformations

**Fig. 10:**
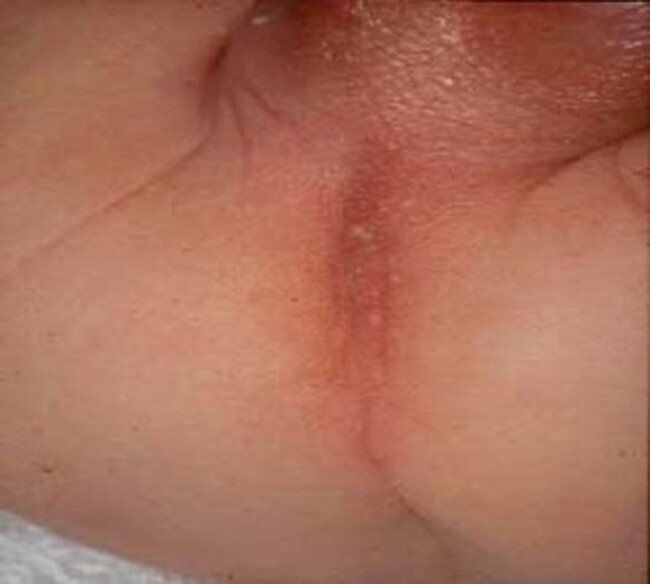
Male newborn with a high anorectal malformation

Anorectal malformations are amongst the most common congenital anomalies of the gastrointestinal tract diagnosed in approximately 1 in 3000 live births. They display extremely heterogenous phenotypes ranging from a slightly malpositioned anus to complex anomalies involving the anorectum, urethra and genitalia^193^. Aetiology is multifactorial without a clearly established genetic background despite occasional association with chromosomal defects and known syndromes. Anorectal malformations arise early in fetal development as a result of disrupted division of the primitive cloaca, leading to impaired separation of the genitourinary tract and bowel^194^. Over 65% of patients have associated anomalies mostly involving the spine, spinal cord, kidneys and other parts of the intestine^193^. The clinical diagnosis is usually apparent immediately after birth due to inability to pass meconium and absence of a normal anal opening in the perineum. In occasional patients with mild malformations the diagnosis may be delayed and is made based on constipation or obstructive symptoms. Anorectal malformations can be classified simply as low or high, but the anatomic abnormality may be more accurately described according to the widely used Krickenbeck classification system (*Table 1*^195^ and *Fig. 10*).

Note absence of anal orifice and flattened buttocks.

**Table 1:** Major clinical groups of anorectal malformations (Krickenbeck classification)^195^

Perineal fistula
**Rectourethral fistula**
Prostatic Bulbar Rectovesical fistula Vestibular fistula Cloaca No fistula Anal stenosis

In general, the surgical treatment is dictated by the level at which the rectum terminates. Patients with a perineal or vestibular fistula can be operated on primarily as newborns or infants. If the anal opening is stenotic but surrounded by the external sphincter muscle, they may be amenable to dilatations or simple anoplasty only. Others may receive a diverting colostomy within 48 h and undergo reconstructive surgery several months later after detailed assessment of the type and anatomy of the malformation and associated anomalies. Operative treatment is mainly based on posterior sagittal anorectoplasty (PSARP; pioneered by Alberto Peña), or less popularly, by anterior sagittal anorectoplasty (ASARP), both of which allow the precise separation and correct relocation of the rectum including reconstruction of the neoanus with the external sphincter muscles under direct vision^196^. The use of an intraoperative muscle stimulator is indispensable. A high-lying rectum opening into the prostatic urethra, bladder neck or vagina may require combined transabdominal laparoscopic or open mobilization. Particularly challenging are female cloacal malformations where rectum, vagina and urethra open into a common channel, which exits in the perineum through a single orifice. In some patients with a long common channel (>3 cm), the vagina may not reach the perineum without additional reconstructions such as sigmoid- or ileo-vaginoplasty.

Depending on the severity of the malformation, patients experience variable degrees of long-term morbidity rates in bowel and genitourinary function, and are at risk of decreased sexual health, fertility and QoL^197^. Functional defects may arise secondary to the combined effects of congenital hypoplastic pelvic sphincter musculature, disordered pelvic innervation, sacral hypoplasia, spinal malformations and surgical injury^193,197^.

While the majority of patients with milder malformations achieve faecal continence, most patients with more severe malformations suffer from deficient faecal control such that assurance of social continence requires active bowel management with regular rectal washouts or antegrade colonic enemas through an appendicostomy or cecostomy^193,197^. Regardless of the severity of the malformation, constipation is common and continues into adulthood in a proportion of patients, necessitating regular laxative treatment and in some cases, bowel management programmes^197^. While social constraints and decreased QoL are rare findings in patients with mild malformations, social disability affects a considerable portion of patients, with the more severe anorectal malformations having impaired long-term faecal continence outcomes^185,197^.

Bladder function and urinary continence are usually well preserved and comparable to normal population controls in patients with milder malformations^187,198^. In more complex anorectal malformations, however, prevalence of neurogenic bladder dysfunction and accompanying urinary incontinence is higher and may be permanent in the most severe cases. These patients may empty their bladder with clean intermittent catheterization or through a continent vesicostomy. Permanent urinary incontinence is most common in female patients operated on for cloacal malformations^199^. Associated congenital malformations of the kidneys together with bladder dysfunction and recurrent urinary tract infections may rarely lead to renal insufficiency.

Genital abnormalities are more common in girls, including duplication and agenesis of the vagina and uterus, most frequently encountered in cloacal malformations^200^. A gynaecological examination is considered essential at the time of puberty to ensure unobstructed menstruation and anatomical capacity for sexual intercourse. Vaginal septation and introital stenosis are typical concerns requiring additional interventions at this time point. Dyspareunia, possibly due to vaginal scarring, is a common concern and sexual dysfunction may affect up to 50% of females^198,200^. The majority of women operated on for anorectal malformations can conceive and carry a pregnancy^200,201^. Elective caesarian section, unlike vaginal delivery, avoids the risk of anal sphincter injury and is possibly the safest delivery mode at least for women with operated on cloacal malformation^200^. Erectile dysfunction and retrograde ejaculation may occur in 10–20% of males, especially in association with complex malformations and those with significant sacral dysplasia^198^.

Patients with HSCR and anorectal malformations therefore require continuous structured healthcare follow-up and support throughout childhood and beyond as functional problems can persist well into adulthood and correlate with social disability and decreased QoL^174^. Patients who continue to experience significant psychosocial limitations in the long term highlight the crucial need for robust transitional care arrangements^197^. There is no doubt that additional longitudinal studies are needed, particularly focused on identifying optimal long-term therapeutic and management strategies. Most importantly, wide interdisciplinary collaboration between paediatric and adult colorectal surgeons, gastroenterologists and other pertinent healthcare professionals is essential as these patients transition into adulthood. As mentioned in the previous section, transitional care pathways for anorectal disorders are not widely adopted, and more so, adult care programmes^174,191,192^. The authors are not aware of adult guidelines for patients with anorectal malformations.

## Thoracic

### Congenital lung malformations

**Fig. 11:**
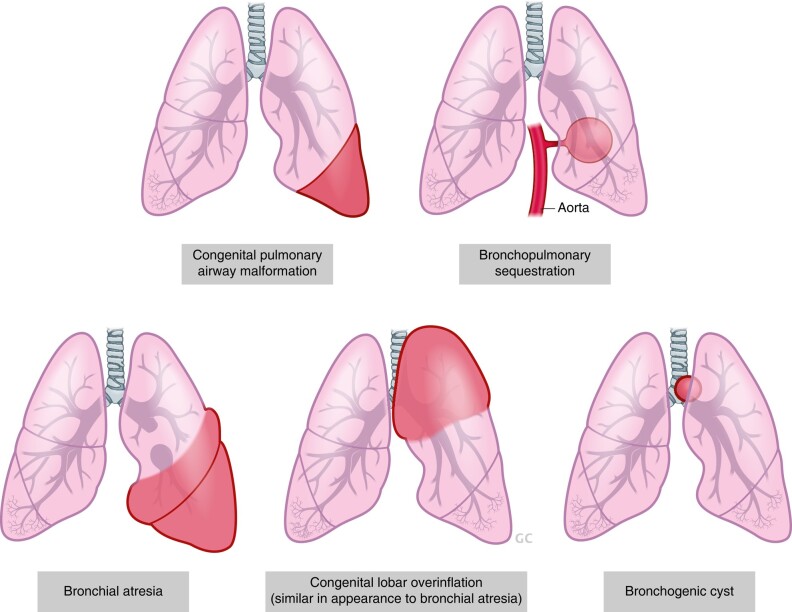
Illustrations of common subtypes of congenital lung lesions

Adapted from Doktor *et al*.^202^.

**Fig. 12:**
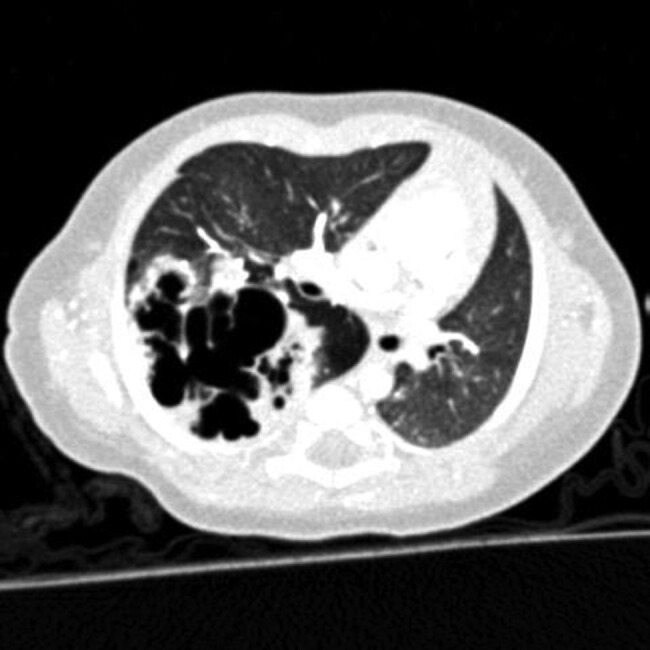
Transverse CT image showing a right lower lobar congenital pulmonary airway malformation (CPAM)

Congenital lung malformations (CLMs) encompass a wide spectrum of developmental disorders. These include congenital pulmonary airway malformation (CPAM), bronchopulmonary sequestration (BPS), bronchial atresia, bronchogenic cysts (BCs) and congenital lobar overinflation (CLO) (*Fig. 11*^202^). Lung lesions were once thought to be very rare, but with recent advances in antenatal diagnosis, the incidence of CPAM, for example, has risen to as high as 1:7200 live births^203^. These abnormalities arise as a result of aberrant organogenesis and dysregulation in epithelial–mesenchymal interaction at various time points during early human lung development^204^. CPAM is the commonest CLM with the lesion in direct communication with adjacent lung parenchyma (*Fig. 12*). It is characterized by proliferation of bronchial structures rather than alveoli. BPS refers to a mass of lung tissue not in continuity with the tracheobronchial tree with an aberrant systemic arterial supply as its key hallmark. On the other hand, CLO is an overinflation of a pulmonary lobe caused by air trapping from bronchial narrowing. In bronchial atresia, there is focal obliteration of a proximal bronchial segment. The lung distal to this becomes overinflated and the bronchi distal to the atresia may appear as a mucocoele on cross-sectional imaging. BC is a unilocular malformation mostly found in the mediastinum adjacent to the trachea or the mainstem bronchi with no direct communication to the tracheobronchial tree.

Whilst the majority of CLM are now diagnosed early in gestation^205^, they mostly cause no detrimental effect, with the fetus often delivered at full term. However, if these lesions are very large they may cause external compression to surrounding structures, leading to pulmonary hypoplasia or hydrops fetalis. The CPAM volume ratio (CVR) is now accepted as an important prognostic index of neonatal outcome in prenatally diagnosed CPAMs^206^. In ‘high-risk’ fetuses with hydrops from large CLM, fetal intervention may be considered. This carries risks of premature delivery and fetal death so parents need to be counselled extensively prior to undertaking fetal surgery^207^. Alternatively and more recently, expedient caesarean section may now be scheduled in specific high-risk fetal cases with emergent postnatal lung lesion resection performed thereafter (‘section-to-resection’) as the preferred strategy option in specialist centres^208^.

After delivery, 90% of babies with CLM are generally asymptomatic, but some may have respiratory distress due to the mass effect of the lesion with mediastinal shift. There is no argument that symptomatic patients will require early/emergency surgery to alleviate symptoms. For asymptomatic patients, there is an unresolved controversy amongst paediatric surgeons about best practice management, currently the subject of an international randomized trial, and also a prospective patient registry^209,210^. Many surgeons worldwide are nonetheless still in favour of prophylactic surgical resection of asymptomatic cases of CPAM, as the key argument for surgery is to prevent future risks of complications, notably recurrent chest infections and malignant transformation^211–213^. To the contrary, some clinicians remain unconvinced of potential risks and consider conservative management with regular follow-up an alternative approach^211–216^. The arguments for a conservative approach centre not only around an overestimation of potential sequelae for the untreated CPAM but also the avoidance of surgery and general anaesthesia in otherwise healthy newborns.

Thoracoscopic lobectomy in children with CLM is considered the standard of care for all its known advantages where feasible^217,218^. Comparing with video-assisted thoracoscopic surgery in adults, thoracoscopic lobectomy in newborns and small infants may only require a single 5 mm and two 3 mm trocars. Recently published data suggest resection of CPAM at a younger age is generally associated with better perioperative outcomes^217,219^.

For patients who have undergone thoracoscopic lobectomy, future risks of recurrent infections and malignancy should be largely eliminated. In terms of parental concerns about lung function after resection of a single lobe, there appears not to be any measurable deficit in mid-term outcomes^220^. Minimally invasive surgical techniques also have more favourable outcomes in offsetting the risk of developing musculoskeletal deformity^221^. In terms of daily activities, exercise capacity for CPAM patients who underwent resection in early life was found to be comparable to healthy controls^220^.

On the other hand, for those patients who are managed non-operatively or undergo a ‘watchful waiting’ policy, the main argument for the need for long-term follow-up is that some children will go on to later develop symptoms of which the most concerning are risks of malignancy, although this metric remains poorly defined. Recent research has shed some light on the association between CPAM and tumours showing the presence of Kirsten rat sarcoma virus (KRAS) gene mutations (one of the most frequently mutated genes in lung cancer) in mucinous and non-mucinous cells of CPAMs type 1^222,223^. Conservative management is typified by structured, regular outpatient visits. Given that most asymptomatic lesions do not significantly change, nor do they appear readily visible on plain chest X-ray, the use of this modality planning is doubtful. CT scan surveillance will certainly provide definitive detailed information but there is a concern of subjecting young children to excessive radiation dosage from repeated CT imaging. Magnetic resonance imaging negates this risk, but is a more limited resource and in childhood often requires general anaesthesia. When these non-operated children reach adulthood the vexing questions that adult surgeons frequently have relate to the length and absolute necessity of long-term follow-up. There have indeed been isolated case reports of lung adenocarcinoma arising from CPAM in adult patients^224–227^. Overall, current literature on long-term outcomes in CLM patients remains scarce, but taking the potential morbidity rate of recurrent respiratory infections, reduced exercise tolerance and risk of malignancies later in life into account, patients should ideally be followed up by specialists in dedicated centres to ensure smooth transitional care into adulthood. With a lack of consensus in best practice management of CLM in childhood, the authors are not aware of any specific or robust recommendations published for the transitional care or adult follow-up of these patients.

### Congenital diaphragmatic hernia

**Fig. 13:**
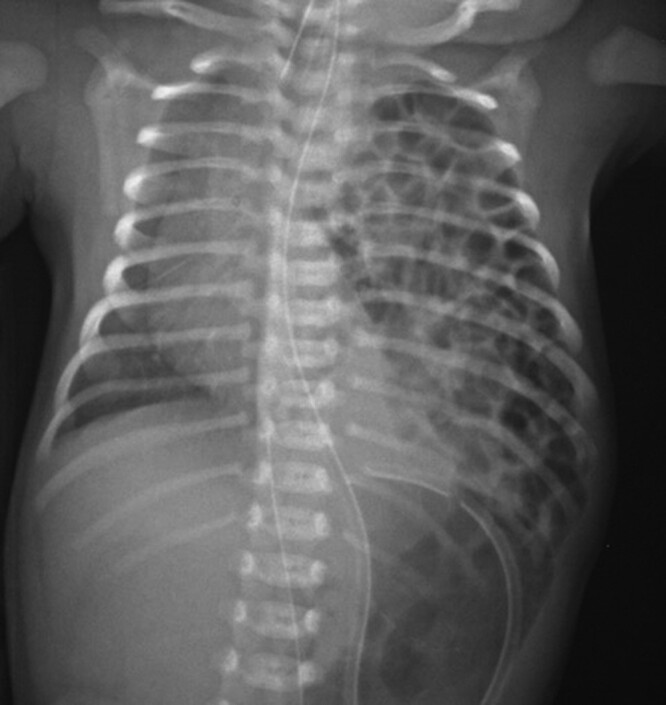
Plain radiograph of a newborn with left congenital diaphragmatic hernia (CDH)

**Fig. 14:**
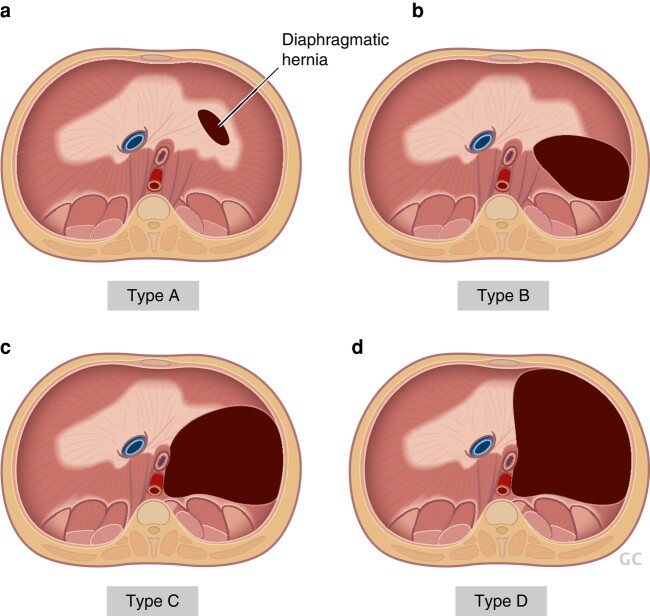
Congenital diaphragmatic hernia (CDH) study group classification system popular for grading severity of diaphragmatic hernia

CDH occurs in 1:2500 births with a baby born approximately every day in the UK (*Fig. 13*)^228^. A defect in closure of the developing diaphragm allows abdominal viscera including liver and spleen to herniate into the thorax, negatively impacting lung development. A primary lung insult involving primordial growth is also postulated, giving rise to the now widely accepted ‘Two Hit Hypothesis’^229,230^. CDH carries an appreciably high mortality rate (more than 50%), with the resultant lung hypoplasia and pulmonary hypertension arising from disorganized airway and aberrant vasculature biology. Urgent operative intervention by means of hernia repair was once surgical dogma; however, over recent decades, elective delivery scheduled at specialist centres following *in utero* diagnosis with stabilization of labile cardiopulmonary physiology and delayed operation is nowadays considered paramount to achieve better survival outcomes^231,232^. The operation involves primary native diaphragm repair or securing prosthetic patch closure dependent on defect size (*Fig. 14*)^233,234^. Classical open repair and minimally invasive techniques are deployed with a growing recognition that not all newborns with a CDH are suitable for thoracoscopy, which is best reserved for smaller diaphragmatic defects in low-risk patients, given higher recurrences in larger defects^234–236^. Fetal risk prognostication according to observed to expected lung-head ratio (O/E LHR) and liver herniation has guided and driven the development of fetal surgery RCTs (the tracheal occlusion to accelerate lung growth (TOTAL) trials) with fetoscopic endoluminal tracheal occlusion (FETO) offered at designated specialist fetal medicine centres in an effort to rescue and stimulate prenatal lung growth^237–239^. Permissive hypercapnia, better termed ‘gentle ventilation’, together with extracorporeal membrane oxygenation (ECMO) have seen CDH survival outcomes (more than 75%) improve at many institutions, such that fetal intervention, at time of writing, is considered controversial and the subject of ongoing debate^240^.

Note intestinal viscerae herniated into left hemithorax.

**a** Small defect amenable to primary closure; **b** and **c** moderate defects which may require patch repair; **d** diaphragmatic agenesis mandates patch closure. Adapted from Lally *et al*.^233^.

CDH survivors require lifelong follow-up best coordinated through multidisciplinary clinics led by paediatric surgeons with respiratory physicians, cardiologists and allied healthcare professionals with transition to adult hospital services considered essential as adolescent years advance^241–243^. Health outcomes and QoL are crucially interlinked to several multiple organ systems which include pulmonary, cardiac, gastrointestinal/nutritional, orthopaedic, neurocognitive and executive functioning^241–244^. Hernia recurrence with reoperation on can occur at any age in aftercare follow-up. GORD in newborns, infancy or childhood requiring fundoplication with or without feeding gastrostomy is not uncommon. Pectus chest wall deformity or scoliosis needing surgical correction or use of bracing appliances can also be problematic secondary to open surgery or patch use^243^. Lung function abnormalities characterized by ventilation-perfusion mismatch involves respiratory medicine aftercare surveillance whilst pulmonary hypertension and cardiac disease mandates a structured healthcare follow-up plan^241–246^. Advocacy and charitable support groups such as CDH UK and CDH International are a vital network portal for many patients and families worldwide from initial antenatal diagnosis extending well beyond into adulthood^247^. Whilst there is a growing interest in the long-term impact of CDH in adult survivors, the authors are not aware of any specific recommendations for transitional care, nor adult care programmes^248^.

## Cancer

**Fig. 15:**
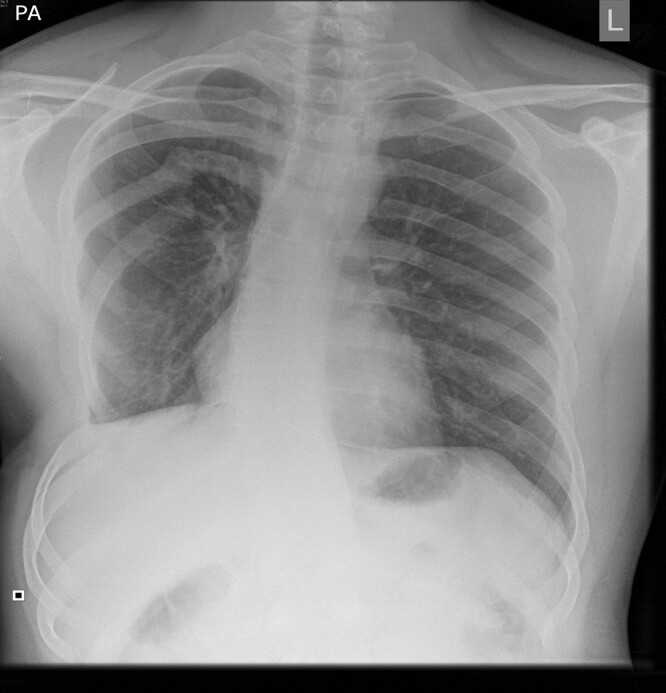
Scoliosis which has developed after chest wall tumour resection

PA, posteroanterior; L, left.

Cancer is the second leading cause of death after trauma in the paediatric age group^249^. Unlike many adult cancers integrally linked to risk lifestyle such as smoking and excess alcohol consumption, paediatric solid tumours often have a developmental biology or genetic component as their basis. Remarkable advances in survival have occurred in recent decades with multimodal cancer therapies. Childhood solid tumour malignancies such as Wilm’s tumour and neuroblastoma have 5-year survival rates now approaching 92 and 60% respectively^250–252^. Success steadily continues with many other solid tumours, notably germ cell tumours and sarcomas, whilst other rare tumours such as aggressive primitive neuroectodermal neoplasms as a key noteworthy example may have variably poor outcomes, with less than 40% survival despite current therapies^253–257^. The defining role of surgery in the curative treatment of many childhood tumours links with individual patient risk staging categorization and biology. Organ-sparing surgery is now steadily applicable to certain Wilm’s tumour (that is ‘nephron-sparing’ operations), benign ovarian and testicular neoplasms, all in a concerted effort to offset consequences of late-onset hyperperfusion-induced renal failure in the formerly nephrectomized patient with a remaining solitary healthy kidney and the lifetime risk of infertility in those harbouring gonadal tumours^258–262^. Surveillance of the long-term effects related to treatment of childhood cancer includes actively monitoring risk of tumour recurrence, metachronous tumour, second late ‘new malignancies’, for example thyroid cancers resultant from earlier primary cancer therapies, chest wall or spinal deformity (*Fig. 15*), growth impairment, functional sequelae and sterility^249,263–265^. Late effects of cancer management in childhood therefore extend into adulthood. For patients with inherited polyposis disorders, such as Peutz–Jeghers syndrome, and family cancer predisposition syndromes, such as Li-Fraumeni syndrome, lifelong follow-up is also critical as multiple new tumours invariably occur in these vulnerable high-risk individuals^266,267^. Functional sequelae resulting from childhood cancer surgery are currently being investigated in an international multicentre collaborative study (FUSE (Functional Sequelae Assessment In Pediatric Surgical Oncology)) led by St Jude Children’s Research Hospital Memphis, USA, and the International Society of Paediatric Surgical Oncology. Transition to adult cancer services is therefore key and essential for long-term follow-up, active monitoring of survivorship health and crucially maintaining QoL^268^.

## Future directions

Over the past few decades, the focus of many surgical conditions of childhood has evolved from a desire to achieve high rates of survival, to understanding the impact on the child and their family, and minimizing the lifelong implications of the pathology and its treatment. With this shift in focus, we will eventually better understand the natural history of some of these conditions (for example congenital lung lesions and biliary atresia), the optimal surgical strategies for affected children (for example Hirschsprung disease and oesophageal atresia) and reduce the long-term morbidity rate from treatment (for example paediatric solid tumours and minimally invasive surgery (MIS) techniques). Historically, high-quality research has been limited by the challenge of consent in patients who often lack capacity, ethical concerns and the rarity of conditions, limiting the volume of single-centre experiences^269^. Routine collection of better data in healthcare, development of core outcome sets, creation of prospective registries, multicentre trials, more translational research and better international collaboration through the development of networks (such as the European Reference Networks in continental Europe), will offer opportunities to achieve these aims. In addition, the increasing survivorship of children affected by surgical conditions will translate into a growing population of adults with lifelong conditions and specialist healthcare needs. The importance of transition from childhood to adulthood is becoming realized^270^. These programmes are currently sparse and in their infancy, but routine transition of care, anticipatory planning and structured follow-up for conditions with lifelong implications need to be developed and made available. Not only do the conditions discussed in this review affect physical health, they carry a psychosocial burden which must be considered by healthcare professionals during long-term follow-up. Studies of adult survivors so far have focused on clinical outcomes, but there is a need and desire for more study and reporting of outcomes most important to patients. Research focusing on the subtleties missed by QoL measurement tools are warranted, to include impact on mental, social, sexual, educational and occupational aspects of wellbeing^271–274^. Furthermore, another key issue that adolescent and young adult patients experience is a lack of familiarity of adult healthcare professionals with rare congenital conditions. Currently, patient support groups remain a useful source of information, provide advocacy and allow those affected by surgical conditions of childhood to network with others (for example TOFS, CDH UK, Reach and Max’s Trust). Patients, however, crucially need adult clinicians to develop interest and expertise in managing surgical conditions of childhood. We hope that the reader finds this article a helpful summary of these rare diseases and their long-term impact for adult surgeons, and that some are inspired to care for these unique, grown-up children.

## Data Availability

This article is based on published literature as a narrative review and therefore there are no data to share separately.
